# Influence of arrival weight of Holstein steers of similar age on feedlot growth performance, dietary energetics, and carcass characteristics

**DOI:** 10.5455/javar.2022.i569

**Published:** 2022-01-16

**Authors:** Rodrigo Flores, Alejandro Plascencia, Alberto Barreras, Jaime Salinas-Chavira, Noemí Torrentera, Richard A. Zinn

**Affiliations:** 1 Instituto de Investigaciones en Ciencias Veterinarias, Universidad Autónoma de Baja California, Mexicali, México; 2 Departamento de Ciencias Naturales y Exactas, Universidad Autónoma de Occidente, Guasave, México; 3 Departamento de Nutrición Animal, Facultad de Medicina Veterinaria y Zootecnia, Universidad Autónoma de Tamaulipas, Cd Victoria, México; 4 Department Animal Science, University of California, Davis 95616, CA, USA; † These two authors contributed equally.

**Keywords:** Arrival weight, feedlot, Holstein, performance, carcass

## Abstract

**Objective:**

Evaluate the effect of arrival weight on feedlot growth performance and carcass characteristics of Holstein steers of similar age.

**Material and Methods:**

Three hundred calf-fed Holstein steers (age 113 ± 1-day) were distributed in a completely randomly unbalanced design and divided into five categories (105, 112, 117, 123, and 129 kg) of shrunk initial weight (SIW). Calves were weighed on days 1, 112, 224, and 361. Calves were fed steam-flaked corn-based diets. Growth performance and dietary energy were evaluated for each period and the study as a whole (1–361-day).

**Results:**

During the rearing period, average daily gain (ADG) increased (linearly effect, *p* < 0.01) with increasing birth weight. Birthweight was positively associated (*p* < 0.05) with feedlot arrival weight (*R*^2^ = 0.47) and final harvest weight (*R*^2^ = 0.36). Overall ADG increased (*p* < 0.01) with increasing SIW. Dry matter intake increased linearly during the first 224-day but quadratically during the last 137 days. Overall, there was a quadratic effect (*p* < 0.05) of SIW on gain-to-feed and observed-to-expected dietary NE, with lower efficiencies (4%) for steers in both the lightest and heaviest SIW. Hot carcass weight, Longissimus muscle area, marbling score, and fat thickness increased (linear effect, *p* ≤ 0.03) as SIW increased, whereas kidney-pelvic-heart fat and yield-grade were unaffected.

**Conclusions:**

The initial arrival weight influences the growth performance, energetic efficiency, and carcass characteristics of Holstein steers of similar age. The effect is more pronounced in the lighter (<112 kg) steers.

## Introduction

Cattle performance prediction in feedlot is a critical decision-making tool for optimal planning of cattle fattening. In this sense, it has been reported that both initial arrival weight and age influence feedlot growth performance [1]

## Materials and Methods

### Ethical approval

Animal care and management were approved by the University of California, Davis, Animal Use and Care Committee (protocol # 20548).

### Animals, diets, and estimation of dietary net energy

A 361-day experiment with 300 Holstein calves was conducted to determine the effect of arrival weight at similar ages on feedlot growth performance, dietary net energy, and carcass characteristics. Steers were vaccinated against bovine rhinotracheitis-parainfluenza (Cattle Master Gold FP 5 L5, Zoetis, New York, NY), clostridials (Ultrabac-7, Zoetis, New York, NY), treated against internal and external parasites (Dectomax Injectable, Zoetis, New York, NY), subcutaneously injected with 1,500 IU vitamin E (as d-alpha-tocopherol) 500,000 IU vitamin A (as retinyl-palmitate) and 50,000 IU vitamin D3 (Vital E-A + D3, Stuart Products, Bedford, TX), and 2.4 gm oxytetracycline (LA-200, Zoetis, New York, NY), branded, and ear-tagged. Steers were blocked by initial shrunk (off truck) initial weight (SIW) into five weight groupings from 105 to 129 kg of SIW. The treatment groups were: 105, 112, 117, 123, and 129 kg of SIW. Animals were randomly assigned within weight groupings to 50 pens, 6 steers/pen. Pens were 78 with 33 m^2^ of overhead shade, automatic waterers, and fence-line feed bunks. Calves were fed with steam-flaked corn-based diets. The receiving diet [14.4% CP, 18.84% NDF, and 2.21 Mcal net energy of maintenance (NE_m_)/kg DM] was provided during the initial 112-day on feed. From day 112 until harvest, all steers received the finishing diet (14.3% CP, 15.37% NDF, and 2.27 Mcal NE_m_/kg DM). Diets were prepared at weekly intervals and stored in plywood boxes located in front of each pen. Steers were allowed *ad libitum* access to feed, provided twice daily. On days 120 and 224, all steers were injected subcutaneously with 500,000 IU vitamin A and implanted with Revalor-S (Intervet, Millsboro, DE).

Dietary net energy estimations were performed based on the averages of shrunk weight, daily weight gain, and DMI of each fattening period following the equations and energetic derivations exposed by Plascencia and Zinn [6].

### Carcass data

Hot carcass weights (HCW) were obtained at the time of slaughter. After carcasses chilled for 48 h, the following measurements were obtained: longissimus muscle (LM) area (cm2) by direct grid reading of the LM at the 12th rib; subcutaneous fat (cm) over the LM at the 12th rib taken at a location 3/4 the lateral length from the chine bone end (adjusted by eye for unusual fat distribution); kidney-pelvic-heart fat (KPH) as a percentage of HCW; marbling score using 3.0 as minimum slight, 4.0 as minimum small, 5.0 as minimum modest, 6.0 as minimum moderate, etc. [7], and estimated retail yield (expressed as % of HCW) of boneless, closely trimmed retail cuts from the round, loin, rib and chuck according to the equation: Retail yield, % of HCW = 52.56 − 1.95 × subcutaneous fat − 1.06 × KPH + 0.106 × LM area − 0.018 × HCW.

### Statistical design and analysis

For calculating steer performance, interim and final live weight (LW) were reduced by 4% to account for digestive tract fill. Pens were used as experimental units. The experimental data were analyzed as completely randomly unbalanced design according to the following statistical model: Yij = μ + Wi + εij, where μ is the common experimental effect, Wi represents initial weight effect (df = 4), and εij represents the residual error (df = 45). Treatments effects were tested using the orthogonal polynomials linear and quadratic. A significant effect was considered at p < 0.05. All statistical procedures were performed using the statistical Statistical Analytical System software [8].

## Results and Discussion

Morbidity and mortality were low (4.3% and 1.0%, respectively) and not affected by weight grouping (*p* > 0.20). The influence of LW at birth on growth performance during the rearing phase is shown in Table 1. During the rearing period, ADG increased (linear effect, *p* < 0.01) with increasing birth weight. Birthweight was positively associated with feedlot arrival weight (*R*^2^ = 0.47, *p* < 0.01) and final harvest weight (*R*^2^ = 0.36, *p* < 0.05). Supporting our results, Maccari et al. [9] observed an ADG of 0.692 kg/day for Holstein calves during the rearing phase (average birth weight 44 kg). Likewise, Bailey and Mears [10] observed a positive correlation between the birth weight of Holstein calves and ADG from birth to weaning (100 kg), accounting for between 24% and 42% of the variation in preweaning ADG. Although to a lesser extent, they also observed an association between birth weight and both rate (*r*^2^ = 0.21) and efficiency (*r*^2 ^= −0.30) of post-weaning ADG. Furthermore, it has been observed that final weaning weight increased by 1.53 kg with every 1 kg increase in birth weight [11]. Similarly, Greenwood et al. [12] observed that lower birth-weight calves and calves that had slower birth to weaning ADG had poorer growth performance during the feedlot phase compared with high birth weight and rapid preweaning growth calves when evaluating the long-term effects of birth weight and growth to weaning on feedlot performance and carcass yield of Piedmontese and Wagyu-cross cattle. These results confirm that pre-weaning weight affects dairy cattle, likewise crossbred beef cattle. The influence of feedlot arrival weight on growth performance and carcass characteristics are shown in Tables 1 and 2. Overall (361-day) ADG increased (linear effect, *p* < 0.01) with increasing arrival weight. Likewise, Salinas-Chavira et al. [13] and Cano et al. [14] observed greater ADG for calf-fed Holstein steers with heavier versus lighter initial arrival weight. As much as all steers were of similar age (113 to 114-day), differences in arrival weight function both birth weight and ADG during the rearing phase. This study did not assess the extent to which these two factors relate to the frame. However, as a breed, the frame size of Holstein steers can be quite variable, with an observed range of between 7.6 and 9.3 [15]. Differences in frame size are directly related to differences in mature size [16] and ADG [17].

**Table 1.** Weight at birth, weight gain before arrival (preweaning and weaning phase), age at the arrival of feedlot, and the influence of the arrival weight on feedlot growth performance and dietary energetics of Holstein calves.

	Initial body weight (Kg)						Effects	
Item	105	112	117	123	129	SEM	Linear	Quadratic
Calves	28	87	79	58	17			
Birth weight, kg	41.7	41.9	43.4	43.6	44.8			
Before arrival ADG, kg/d	0.559	0.611	0.649	0.695	0.737	0.01	<0.01	0.60
Age at shipment, day	113	114	114	114	113	0.63	0.97	0.54
Arrival LW, kg	105	112	117	123	129	0.75	<0.01	0.99
Pen replicates	5	15	15	10	5			
Birth LW, kg	41.7	41.9	43.4	43.6	44.8	1.0	<0.01	0.98
LW, kg a	105	112	117	123	129	0.75	<0.01	0.99
112-day	250.3	264.8	271.6	282.3	289.9	3.4	<0.01	0.38
224-day	413.2	431.6	439.6	445.5	458.1	5.6	<0.01	0.45
Final	583.7	616.8	623.7	614.5	639.8	10.1	0.01	0.30
ADG, kg								
1–112-day	1.30	1.37	1.38	1.43	1.44	0.03	<0.01	0.36
112–224-day	1.44	1.48	1.49	1.44	1.49	0.04	0.49	0.75
224–361-day	1.23	1.33	1.36	1.27	1.37	0.04	0.06	0.26
1–361-day	1.31	1.39	1.41	1.37	1.43	0.03	<0.01	0.27
DMI, kg								
1–112-day	4.79	4.96	5.09	5.23	5.35	0.08	<0.01	0.76
112–224-day	7.19	7.34	7.49	7.44	7.84	0.15	<0.01	0.44
224–361-day	9.38	9.29	9.31	9.18	10.16	0.12	<0.01	<0.01
1–361-day	7.29	7.35	7.43	7.39	7.93	0.09	<0.01	<0.01
ADG/DMI								
1–112-day	0.271	0.276	0.271	0.273	0.269	0.004	0.55	0.36
112–224-day	0.201	0.201	0.199	0.194	0.190	0.005	0.03	0.34
224–361-day	0.131	0.143	0.147	0.138	0.134	0.004	0.77	<0.01
1–361 day	0.180	0.189	0.189	0.186	0.180	0.003	0.68	<0.01
Observed-to-expected NEm								
1–112-day	0.904	0.936	0.939	0.959	0.966	0.011	<0.01	0.33
112–224-day	0.981	1.008	1.009	1.012	1.001	0.017	0.33	0.15
224–361-day	0.931	1.013	1.033	1.006	0.979	0.021	0.10	<0.01
1–361 day	0.950	1.007	1.018	1.005	0.992	0.015	0.05	<0.01
Observed-to-expected NEg								
1–112-day	0.878	0.919	0.922	0.948	0.956	0.014	<0.01	0.33
112–224-day	0.976	1.01	1.012	1.015	1.002	0.022	0.33	0.15
224–361-day	0.914	1.017	1.043	1.008	0.973	0.026	0.10	<0.01
1–361-day	0.937	1.009	1.022	1.006	0.990	0.019	0.05	<0.01

**Figure 1.**
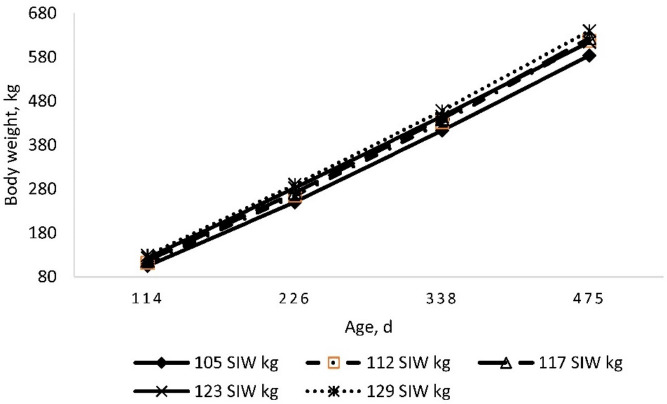
Body weight with respect to the age of calf-fed Holstein steers. All steers were similar age at the beginning of the study (113 ± 1 day).

**Figure 2.**
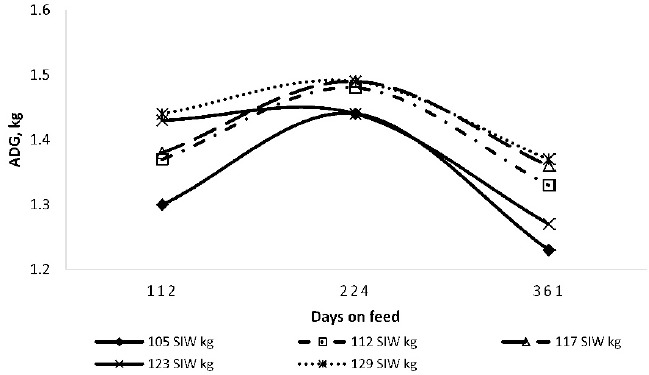
ADG with respect of days on feed of Calf-fed Holstein steers.

The relationship between age and body weight of Holstein calf-steers during the study is shown in Figure 1. Steers within the higher SIW grouping (129 kg) were heavier throughout the study, whereas steers in the lighter SIW grouping (105 kg) were lighter throughout the study (Fig. 2). Consistent with a previous study [13], ADG among steer groupings was greater during the first and second 112-day feeding periods (65.0% and 39.9%, respectively) than during the final period (224–361 day) of the study. As expected, differences in ADG reflected greater relative DMI (as % LW) during the initial two 112-day periods versus the final period (31.3% and 14.6% greater intake as a proportion of LW, respectively).

**Figure 3.**
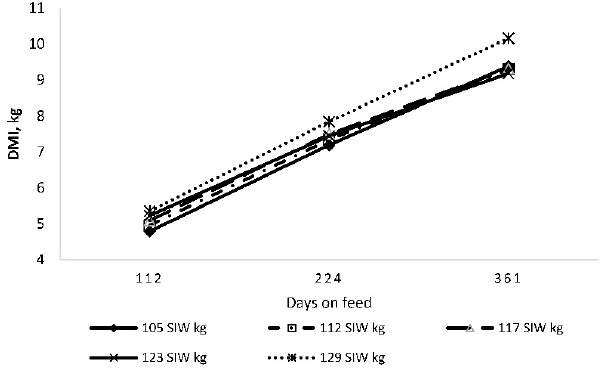
DMI with respect of days on feed of Calf-fed Holstein steers.

DMI increased as initial body weight increased. The effect was linear (p < 0.01) until day 224 and for the overall feeding period (1–361 day). There was a quadratic effect (p < 0.01) of initial weight on DMI during the final period (last 137 of the study), with the heavier weight grouping consuming 7.8% more DM than the average of the other groupings. The overall increase in DMI with increasing initial weight is consistent with concomitant increases in overall ADG. Salinas-Chavira et al. [13] divided 144 newly received Holstein steers into two body weight groupings (lighter-half, averaging 117 kg and heavier-half, averaging 121 kg). Consistent with the present study, they observed that although the difference in average initial weight of the two groupings was only 5 kg, the heavier grouping had greater overall (340-day) DMI associated with greater ADG. According to NASEM [18], initial LW accounts for 60% of the variation in DMI among feedlot cattle within sex and frame size. Due to the protracted nature of the feeding period of Holstein steers in the feedlot, relatively small differences in DMI and hence ADG result in appreciably significant differences in final carcass weight. Based on the present study, a primary contributing factor was associated with the DMI and associated ADG of the lighter initial weight grouping (representing 10% of total cattle received). The relationship between days on feed and DMI is shown in Figure 3. Similar to previous reports [2,13], trends in DMI reflect differences in initial weight.

**Table 2.** Influence of arrival weight on carcass traits of Holstein steers.

	Initial LW, kg						Effects	
Item	105	112	117	123	129	SEM	Linear	Quadratic
HCW, kg	363.1	383.6	387.9	382.2	397.9	6.26	<0.01	0.30
Dressing percentage	61.7	62.4	62.5	62.2	61.5	0.34	0.61	<0.01
KPH, %	2.4	2.4	2.4	2.4	2.5	0.16	0.70	0.47
Fat thickness, cm	0.56	0.68	0.63	0.66	0.74	0.06	0.03	0.91
Longissimus area, cm2	77.6	77.6	85.1	85.2	88.1	2.14	<0.01	0.87
Marbling score	4.4	5.5	5.5	5	6.5	0.31	<0.01	0.62
Retail yield, %	50.4	49.7	50.6	50.7	50.5	0.29	0.19	0.84
Yield grade	2.7	3.0	2.6	2.6	2.7	0.12	0.16	0.86

Weight groupings did not affect the gain-to-feed ratio (G:F) during the initial 112-day periods. G:F decreased during the second 112-day period (linear effect, p < 0.05) as initial body weight increased. During the final 137-day period and overall (1–361 day), there was a quadratic effect (p < 0.05) of initial weight grouping on G:F, with efficiency being poorer (4%) for steers in both the lightest and heaviest weight groupings. This is in disagreement with the findings of Salinas-Chavira et al. [13] since they noticed a greater G:F for heavier versus lighter Holstein steers. However, they noted that the effect was more pronounced during the late finishing phase.

The overall (361-day) estimated dietary NE based on growth performance was in narrow accordance with NE expected value according to diet formulation. The ratio of observed-to-expected dietary NE increased as initial body weight increased during the first 112-day (linear effect, p < 0.01). During the late finishing phase and overall, there was a quadratic effect (p < 0.01) of initial weight grouping on the ratio of observed-to-expected, being maximal for steers in the 117 kg weight grouping. Whereas the overall lower efficiency of energy utilization during the initial 112-day period is a reflection of dietary deficiencies of limiting essential amino acids in this early stage of growth [19]; thus, the quadratic component to overall efficiency (361-day) is again mainly attributable to poorer performance and efficiency of steers in the lightest weight grouping.

Treatment effects on carcass characteristics are shown in Table 1. Shrunk final weight, HCW, LM area, marbling score, and fat thickness increased (linear effect, p ≤ 0.03) as increased SIW. The initial weight grouping influenced kidney pelvis heart fat and yield grade (p > 0.10). Due to the difference in the rate of gain between lighter versus heavier cattle, it is expected that the heavier group had greater HCW and LM area. Likewise, Salinas-Chavira et al. [13] observed greater HCW and LM area in heavier versus lighter Holstein steer groupings.

## Conclusion

The initial arrival feedlot weight distribution influences growth performance, dietary energetic efficiency, and carcass characteristics of Holstein calves of similar age. Therefore, a pre-weaning gain can be an essential factor in predicting the performance of Holstein calves in the feedlot. The effect was more pronounced in the lighter (<112 kg LW) calves.

## List of Abbreviations

DMI, dry matter intake; d, day; h, hour; ADG, average daily gain; SIW, shrunk initial weight; LW, live weight; NEg
